# Metabolic Signaling Meets Epigenetic Regulation: How Protein Lactylation Remodels the Tumor Immune Microenvironment in Gastric Cancer

**DOI:** 10.3390/cimb48060595

**Published:** 2026-06-04

**Authors:** Xiaoxuan Pan, Xin Chen, Chunyuan Zhang, Xin Ma, Jieru Han

**Affiliations:** 1 Second Clinical Medical College, Heilongjiang University of Chinese Medicine, Harbin 150040, China; panxiaoxuan2005@126.com; 2 First Clinical Medical College, Heilongjiang University of Chinese Medicine, Harbin 150040, China; radinen@outlook.com (X.C.); 18009333891@163.com (C.Z.); 3 Qingdao Medical College of Qingdao University, Qingdao University, Qingdao 266000, China; maxin_0508@163.com; 4 School of Basic Medical Sciences, Heilongjiang University of Chinese Medicine, Harbin 150040, China

**Keywords:** protein lactylation, gastric cancer, metabolic reprogramming, tumor immune microenvironment, lactate, epigenetic regulation, warburg effect, tumor-associated macrophages

## Abstract

This review argues that protein lactylation—a lactate-driven posttranslational modification—serves as the long-sought molecular bridge that coordinates these two hallmarks in gastric cancer (GC). Far from being a passive metabolic byproduct, lactylation operates as a central molecular hub with a dual function: intracellularly, it directly drives malignant phenotypes by modifying key oncoproteins such as YAP and metabolic enzymes; extracellularly, it remodels the tumor immune microenvironment by polarizing tumor-associated macrophages toward an immunosuppressive M2 phenotype, upregulating PD-L1 expression, and impairing CD8^+^ T-cell function. We propose that these two arms constitute a self-reinforcing metabolic–epigenetic–immunological circuit, wherein lactylation both originates from and perpetuates the Warburg effect, creating a vicious cycle that sustains malignancy and immune evasion. This framework positions lactylation not merely as a mechanistic detail, but as a unifying principle that integrates metabolic reprogramming, epigenetic regulation, and immune suppression in GC. We critically evaluate the current landscape of lactylation “writers,” “erasers,” and “readers”; highlight the translational potential of targeting this pathway; and identify the conceptual and technical bottlenecks that must be overcome—including the lack of causality in current studies, the absence of specific research tools, and the unresolved heterogeneity of lactylation across cell types and disease stages. By reframing lactylation as an actionable hub rather than a downstream consequence, this review provides a roadmap for advancing lactylation-based precision medicine in GC.

## 1. Introduction

GC remains one of the leading causes of cancer-related mortality worldwide, with high incidence and mortality rates underpinned by complex pathogenesis and significant therapeutic challenges, including drug resistance and frequent recurrence [1,2]. While conventional treatments such as surgery, chemotherapy, and radiotherapy offer some benefit, their efficacy is often limited [3]. Over the past decade, it has become increasingly clear that tumor cells do not act in isolation; rather, they exist within a complex ecosystem where metabolic reprogramming and immune microenvironment remodeling are intimately intertwined [4]. Yet, a fundamental question has persisted: What molecular mechanism allows cancer cells to simultaneously rewire their own metabolism and reshape the surrounding immune landscape in a coordinated manner?

GC cells exhibit significant metabolic heterogeneity, yet a hallmark feature prevalent among them is the “Warburg effect”—a preferential reliance on aerobic glycolysis even under oxygen-sufficient conditions [5,6]. This metabolic switch facilitates rapid energy production and abundant biosynthesis, supporting uncontrolled proliferation and survival [7,8]. Consequently, glucose is avidly consumed, leading to the aberrant accumulation of its end product, lactate, within tumor cells and the surrounding microenvironment [9].

Historically, lactate was viewed as little more than a metabolic waste product or a contributor to acidic microenvironmental stress, promoting tumor invasion and suppressing immune cell function [10]. This perspective, however, failed to explain how a single molecule could orchestrate such diverse and coordinated effects across both cancer cells and immune cells. The landmark discovery of protein lactylation in 2019 fundamentally changed this view: researchers first reported that lactate could directly serve as an acyl donor, covalently modifying histone lysine residues to form a novel posttranslational modification, protein lactylation (Kla) [11,12]. Lactate, it turned out, is not merely excreted but can be directly “written” into the epigenetic code, functioning as a “metabolite signaling molecule” capable of translating real-time metabolic flux into stable changes in gene expression and cellular function [13,14,15].

In GC, this concept takes on particular significance. First, GC is characterized by an exceptionally active Warburg effect, driven by key enzymes such as PKM2 and exacerbated by risk factors such as Helicobacter pylori infection, which substantially enhances glycolysis [7,16,17,18,19]. This creates a rich substrate pool for lactylation modifications. Second, the immune microenvironment of GC is profoundly immunosuppressive, and lactate has been proven to be a key immune regulator [20,21]. The mechanism of lactylation provides a direct molecular bridge explaining how lactate can precisely regulate the function and polarization state of immune cells at the epigenetic level [22,23].

Despite the explosion of studies identifying lactylation events in GC, the field remains fragmented [24,25]. Most investigations focus on either cancer cell-autonomous effects or immune cell modulation, treating them as separate phenomena. In this review, we propose a unifying framework: protein lactylation functions as a central hub that concurrently coordinates malignant progression and immune evasion in GC. We argue that lactylation operates through two mechanistically distinct but functionally synergistic arms—an intrinsic arm that directly fuels cancer cell proliferation, invasion, and therapy resistance, and an extrinsic arm that reprograms the immune microenvironment toward immunosuppression. Crucially, these two arms are not independent; they form a self-reinforcing circuit wherein lactylation-driven metabolic rewiring amplifies lactate production, which in turn sustains further lactylation in both cancer cells and immune cells, creating a vicious cycle that locks the tumor–immune system into a malignant state [26,27,28,29].

This framework has profound implications. It suggests that lactylation is not merely a downstream readout of metabolic activity but an actionable vulnerability—a point where metabolism, epigenetics, and immunity converge. By integrating recent advances in the identification of lactylation “writers,” “erasers,” and “readers” [30,31], and by critically examining the evidence for its dual role in GC, this review aims to provide a conceptual roadmap for future research. We also confront the field’s most pressing challenges: the scarcity of causal evidence, the lack of specific tools to distinguish lactylation from acetylation [32,33], the unresolved heterogeneity of lactylation across cell types, and the translational barriers that must be overcome [34,35,36,37,38,39]. Ultimately, we contend that understanding and targeting lactylation may offer a paradigm shift in how we approach GC—moving from targeting isolated pathways to disrupting the central hub that coordinates malignancy and immune suppression.

## 2. “Writers,” “Erasers,” and “Readers” of Protein Lactylation

### 2.1. “Writers”: Enzymes That Catalyze Protein Lactylation

Protein lactylation, a novel posttranslational modification, requires specific enzymatic machinery to catalyze the transfer of lactyl groups onto target proteins [30,40]. The “writers” of lactylation primarily utilize lactate—the end product of glycolysis—as the substrate for modification [41,42]. Among the key enzymes identified, the histone acetyltransferase p300/CBP has been reported to exhibit lactyltransferase activity, enabling it to transfer lactyl groups to lysine residues on histones and non-histone proteins [43,44,45].

p300/CBP integrates metabolic status into epigenetic outputs by sensing the lactyl-CoA/acetyl-CoA ratio and local cofactor availability. However, alternative lactylation pathways exist (e.g., *AARS1*-mediated and HDAC1-3-catalyzed), indicating a broader enzymatic landscape. Collectively, these findings indicate that p300/CBP integrates cellular metabolic status into distinct epigenetic outputs by sensing the lactyl-CoA/acetyl-CoA ratio and local cofactor availability. Notably, p300/CBP-dependent lactylation is not the only route for protein lactylation; alternative mechanisms exist, such as *AARS1*-mediated lactylation using lactate and ATP directly (discussed below) and HDAC1-3-catalyzed lactylation independent of lactyl-CoA [26,46]. Therefore, the competition between acetylation and lactylation should be interpreted within this broader enzymatic landscape.

In addition to classical epigenetic regulators, certain moonlighting enzymes also serve as lactyltransferases [43,44]. For example, in GC, the alanyl-tRNA synthetase *AARS1*—canonically involved in protein synthesis—acts as a bona fide lactyltransferase that directly uses lactate and ATP to lactylate YAP, thereby activating YAP-TEAD signaling and promoting tumor proliferation [26,47]. These “writer” enzymes effectively convert metabolic lactate into stable epigenetic or functional signals, establishing a critical molecular bridge between metabolic reprogramming and malignant phenotypes in GC [48,49].

Recent studies have further expanded the repertoire of lactylation writers. In addition to p300/CBP, other acetyltransferases, such as GCN5 and PCAF, have also been shown to possess lactyltransferase activity under high-lactate conditions, particularly in the context of inflammatory and hypoxic tumor microenvironments [50]. Moreover, metabolic enzymes themselves can directly participate in lactylation [51]. Lactate dehydrogenase A (*LDHA*), the key enzyme that catalyzes pyruvate-to-lactate conversion, not only produces a substrate but also facilitates local lactylation of histones (e.g., H3K18la) near its metabolic complex, creating a spatially coupled metabolic–epigenetic module [52,53]. In GC, the upregulation of *LDHA* is correlated with increased global lactylation levels and poor prognosis, highlighting its dual metabolic and “writing” role [52,54]. In addition to histones, nonhistone lactylation writers have been identified. For example, the mitochondrial enzyme *SIRT5*, known for its deacetylase and desuccinylase activities, has been reported to exhibit lactyltransferase activity toward several metabolic enzymes, thereby linking lactylation to mitochondrial function and redox balance [55,56]. Additionally, the RNA-binding protein *HNRNPA2B1* can undergo autolactylation under lactate-rich conditions, which enhances its ability to stabilize oncogenic mRNAs in GC cells [57]. These findings collectively demonstrate that the “writer” network for protein lactylation is more diverse and functionally broad than initially recognized, encompassing not only dedicated epigenetic modifiers but also metabolic enzymes and RNA-binding proteins, all of which contribute to the establishment of lactylation-driven oncogenic programs in GC [58,59].

### 2.2. “Erasers”: Enzymes Removing Protein Lactylation

In the dynamic regulation of protein lactylation, “erasers,” or delactylases, play crucial roles [60,61]. They are responsible for catalyzing the removal of lactyl groups from proteins, thereby establishing lactylation as a reversible and precisely regulated posttranslational modification. In GC, several classes of enzymes with delactylase activity have been identified, primarily through loss-of-function and gain-of-function studies [62,63].

Among these, *SIRT1* has been confirmed by multiple studies as a key histone delactylase that specifically removes the lactyl modification from histone H3 lysine 18 (H3K18la) [64]. In GC cells, the overexpression of *SIRT1* significantly reduces H3K18la levels, whereas its knockdown leads to H3K18la accumulation. This, in turn, triggers a positive feedback loop involving lncRNA *H19*, glycolysis, and further lactylation, driving tumor progression [27,65]. This regulatory mechanism is also corroborated in clinical samples, where low expression of *SIRT1* is correlated with poor prognosis in GC patients [27,66].

In addition to *SIRT1*, *SIRT2* has also been demonstrated to be an important delactylase. Research has shown that under copper stress, *SIRT2* directly removes the lactyl modification from lysine 229 (K229) of the *METTL16* protein [67,68]. This delactylation inhibits *METTL16*’s m6A methyltransferase activity, thereby blocking its modification of downstream *FDX1* mRNA and ultimately inhibiting cuproptosis in GC cells [69,70]. This discovery reveals a novel mechanism by which *SIRT2* participates in determining cell death fate through the regulation of nonhistone lactylation [71].

Further research has expanded the variety of delactylases. Class I histone deacetylases (*HDAC1* and *HDAC3*) have also been reported to possess delactylase activity in vitro and within cells and are capable of removing lactyl modifications from histones [72,73]. Although direct studies on the delactylation function of *HDAC1-3* in GC are ongoing, given their central role in epigenetic regulation, they are likely involved in balancing the lactylation network in GC. Additionally, studies suggest that *SIRT3* may also have delactylation potential, enriching the diversity of the lactylation “eraser” family [74,75,76]. These delactylases work in concert with lactyltransferases (“writers”) to precisely regulate the levels of intracellular lactylation modifications. An imbalance in their activity directly influences the malignant phenotype of GC. For example, loss of delactylase function leads to excessive lactylation of oncogenic proteins, promoting dysregulated gene expression, metabolic reprogramming, immune evasion, and therapy resistance [27,69]. Therefore, targeting the restoration or enhancement of delactylase activity has emerged as a highly promising therapeutic strategy for reversing oncogenic lactylation and intervening in GC progression.

### 2.3. “Readers”: Effector Proteins Recognizing and Binding to Lactylation Marks

Lactylation “reader” proteins refer to effector proteins capable of specifically recognizing and binding to lactylation sites on histones or nonhistone proteins. They translate metabolically derived lactylation signals into biological outputs such as transcriptional regulation, alterations in protein function, or cellular signal transduction. Currently, research in this field is still in its early stages, with a limited number of identified “reader” proteins, and their recognition mechanisms and functional networks have not yet been fully elucidated. In GC, the identified “readers” primarily include epigenetic modifying enzymes, signaling kinases, and deubiquitinating enzymes, which play critical roles in tumor progression, immune evasion, and therapeutic resistance by recognizing lactylation marks [77,78].

#### 2.3.1. Recognition Domains and Molecular Basis of Lactylation “Readers”

**Figure 1:**
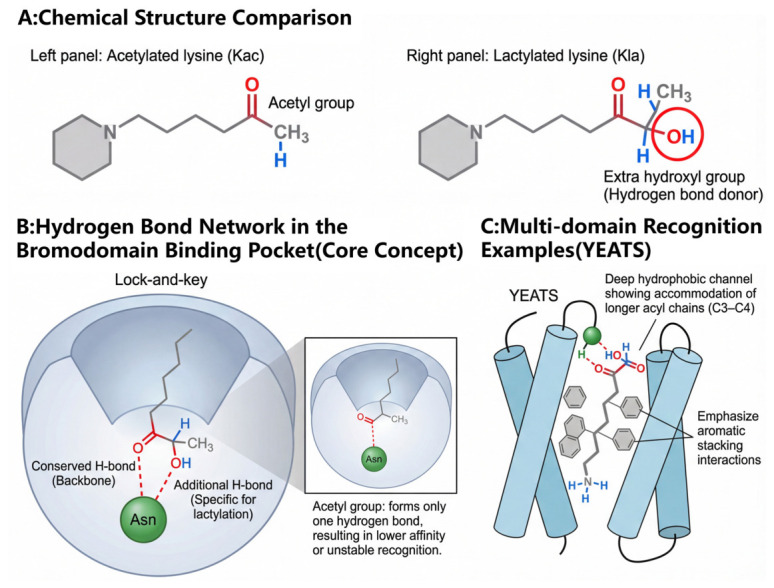
Molecular basis of lactylation recognition. (**A**) Chemical structures of Kac and Kla. The hydroxyl group (-OH) in lactyl moiety provides an additional hydrogen bond donor. (**B**) Proposed bromodomain recognition model. (**C**) Non-bromodomain readers such as SH2 domain recognize lactylated GPX4. All figures were created by the authors using hand-drawing software (including PowerPoint 2023, Adobe Illustrator 2023, and Figdraw online version). No AI-generated images were used.

Currently known lactylation “reader” proteins recognize lactylated lysine (Kla) primarily through specific structural domains. Although lactylation and acetylation are chemically similar (both are acyl modifications), the additional hydroxyl group of the lactyl moiety confers a unique hydrogen bond donor capacity, potentially enabling recognition by certain domains in a manner distinct from acetylation (Figure 1).

① Bromodomain: A Bifunctional Recognition Module for Both Acetylation and Lactylation

The bromodomain is the most classical acetylation recognition domain, and recent studies have found that it can also recognize lactylation. In GC, the acetyltransferase HBO1 has been confirmed to possess lactyltransferase activity, and its bromodomain also acts as a “reader” that specifically recognizes H3K18la. It is recruited to target gene promoter regions, promoting the transcription of cell cycle-related genes and driving GC proliferation [79,80]. Structural biology studies have revealed that the bromodomain of HBO1 recognizes lactylated lysine through a conserved asparagine residue that forms a hydrogen bond network, while a hydrophobic pocket accommodates the alkyl chain of the lactyl group. Its affinity for H3K18la (Kd ≈ 2–5 μM) is comparable to that for H3K18ac, suggesting that bromodomains may function as “multi-acyl” readers involved in signal transduction of various metabolism-derived modifications. Furthermore, the bromodomain of *TRIM33* has also been shown to directly bind H3K18la [81], further supporting the broad role of bromodomains in lactylation recognition.

② SH2 Domain: A Recognition Platform for Nonhistone Lactylation

In addition to histones, nonhistone lactylation can also be recognized by signal transduction proteins. The tyrosine kinases *EGFR* and *SRC* can recognize lactylated *GPX4* through their SH2 domains, increasing *GPX4* stability and inhibiting ferroptosis, thereby promoting the progression of diabetes-associated GC [82,83]. The mechanism may involve electrostatic interactions between positively charged residues in the SH2 domain and the negatively charged carbonyl oxygen of the lactyl group. This discovery expands the scope of lactylation “readers” and suggests that lactylation can directly participate in regulating kinase signaling networks.

In addition, the glycolytic enzyme *PFKM* can interact with H3K18la to promote *CNTN1* transcription and GC cell invasion [84].

#### 2.3.2. Commonalities and Specificities of Lactylation Recognition

**Figure 2:**
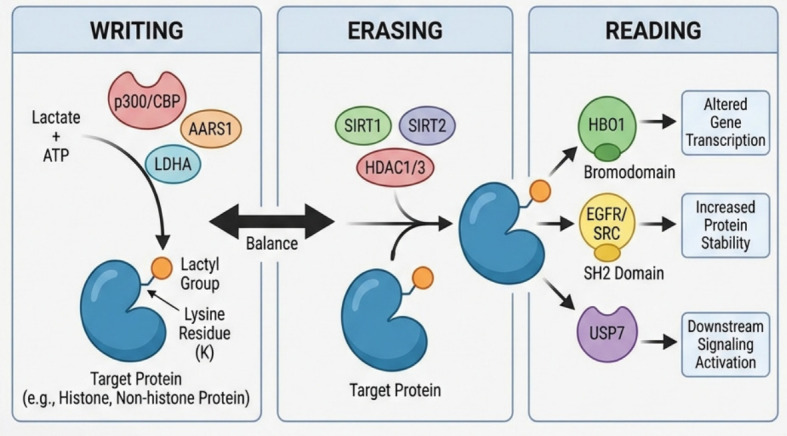
Dynamic Regulatory Machinery of Protein Lactylation: Writers, Erasers, and Readers. This diagram details the enzymatic machinery governing the protein lactylation cycle. Writers (e.g., p300/CBP and *AARS1*) utilize lactate and ATP to catalyze the addition of a lactyl group to lysine residues on substrate proteins. Erasers (e.g., *SIRT1*, *SIRT2*, and HDACs) hydrolytically remove lactyl modifications, ensuring reversibility. Readers (e.g., HBO1 and *EGFR*) contain specific domains that recognize and bind to lactylation marks, transducing the signal into biological outcomes such as altered gene transcription or protein activity. All figures were created by the authors using hand-drawing software (including PowerPoint 2023, Adobe Illustrator 2023, and Figdraw online version). No AI-generated images were used.

Based on the current limited evidence, the recognition characteristics of lactylation “readers” can be preliminarily summarized (Table 1 and Table 2, Figure 2). In terms of chemical basis, most recognition domains bind acyl chains through hydrogen bonding and hydrophobic interactions, while the hydroxyl group of the lactyl moiety may form additional hydrogen bonds, potentially enabling selective recognition by certain domains. Regarding domain types, bromodomains, SH2 domains, and YEATS domains are all known acyl modification recognition modules, but different domains exhibit varying preferences for acyl chain length (C2–C4). In terms of binding affinity, available evidence suggests that Kla and Kac often exhibit comparable affinities for the same reader (e.g., HBO1), but specific domain mutants may achieve selective recognition of Kla. Regarding functional output, recognition typically leads to transcriptional activation, protein stabilization, or signal transduction, while readers that recognize Kla are often associated with metabolic stress and immune remodeling.

## 3. Mechanisms and Roles of Protein Lactylation in Gastric Cancer

### 3.1. Driving Malignant Phenotypes in GC Cells: Proliferation, Invasion, Metastasis, and Therapy Resistance

Protein lactylation has emerged as a key regulator linking tumor metabolism to malignant behavior. Recent research has revealed that lactylation plays a role as a mere metabolic byproduct marker, actively driving the acquisition and maintenance of core malignant phenotypes in GC cells, including dysregulated proliferation, invasion, metastasis, stemness, and therapy resistance. By serving as a substrate for modifying both histone and nonhistone proteins [27], lactate directly reprograms the transcriptomic and signaling networks within cancer cells, acting as an intrinsic engine for GC progression [28,29].

#### 3.1.1. Lactylation-Mediated Activation of Transcription Factors and Oncogenes

Lactylation can directly modify and activate core transcription factors, initiating oncogenic gene expression programs. A prime example is the Hippo pathway effector YAP [89]. Studies have revealed that alanyl-tRNA synthetase 1 (*AARS1*) functions as a noncanonical lactyltransferase, directly utilizing lactate and ATP to catalyze YAP lactylation at specific lysine residues (e.g., K494), bypassing the need for lactyl-CoA [90]. This modification stabilizes YAP and promotes its nuclear translocation [91,92], where it partners with TEAD transcription factors to activate downstream proliferative and prosurvival genes (e.g., *CTGF* and *CYR61*). Clinically, *AARS1* is overexpressed in approximately 68% of primary GC tissues, and its high expression correlates with poor prognosis (hazard ratio [HR] = 2.4) [90]. Functionally, knockdown of *AARS1* decreases GC cell proliferation by 42% in vitro [90]. Furthermore, owing to broader oncogenic principles, lactylation may also stabilize or enhance the activity of other key transcription factors, such as c-Myc, potentially driving cell cycle progression and metabolic reprogramming, representing a significant avenue for future investigations.

#### 3.1.2. Lactylation Modulates RNA Processing and Signaling Pathways

Lactylation also contributes to malignancy by modifying proteins involved in RNA metabolism, thereby aberrantly activating protumorigenic signaling pathways. For example, lactylation of nucleolin (*NCL*) (e.g., at Lys477 by p300) has been reported to influence the alternative splicing of specific mRNAs (e.g., *MADD*), leading to sustained activation of survival pathways such as the MAPK/ERK pathways [93]. In GC, similar mechanisms are likely operative. Lactylation may reshape the posttranscriptional regulatory landscape by modifying RNA-binding proteins, splicing factors, or m6A modification enzymes (e.g., *METTL3/16*), thereby promoting invasion, metastasis, and stress adaptation [93,94].

#### 3.1.3. Metabolic Enzyme Autoregulation and the Vicious Cycle

A self-reinforcing positive feedback loop between protein lactylation and tumor metabolism is pivotal for sustaining malignant phenotypes. Key glycolytic proteins themselves are targets of lactylation. For example, the overexpression of glucose transporter 3 (GLUT3) in GC upregulates lactate dehydrogenase A (*LDHA*), increasing lactate production and overall lactylation levels (e.g., H3K18la) [29]. Conversely, elevated lactylation may further modify metabolic enzymes such as PKM2 or *LDHA*, altering their activity or stability and perpetually amplifying glycolytic flux. This “metabolic reprogramming → lactate production → protein lactylation → further metabolic reprogramming” cycle constitutes a powerful oncogenic engine, maintaining cancer cells in a highly proliferative and invasive state [28,29]. Notably, histone H3K18 lactylation (H3K18la) not only participates in this metabolic feedback loop within cancer cells but also plays a critical role in reprogramming tumor-associated macrophages; the latter will be detailed in Section 3.2.

#### 3.1.4. Lactylation and Therapeutic Resistance

The aberrant activation of oncogenic pathways driven by lactylation directly contributes to resistance to conventional therapies. Research indicates that lactylation-related gene signatures can predict chemotherapy sensitivity. GC cells with high lactylation scores exhibit different half-maximal inhibitory concentrations (IC50) for agents such as cisplatin and 5-fluorouracil, suggesting an association between lactylation levels and chemoresistance [95]. Underlying mechanisms may involve lactylation-mediated activation of survival pathways (e.g., YAP and AKT-mTOR), enhanced DNA damage repair, or apoptosis evasion. Consequently, targeting key lactylation enzymes (e.g., *AARS1* and *LDHA*) or activating delactylases (e.g., SIRT1) is considered a promising strategy to reverse this form of therapeutic resistance [26,27].

In summary, protein lactylation actively and directly reprograms the epigenetic, signaling, and metabolic networks within GC cells by modifying transcription factors (e.g., YAP), RNA metabolic proteins, and core metabolic enzymes. It not only directly activates central oncogenic pathways that drive proliferation and invasion but also establishes a self-sustaining metabolism–epigenetics feedback loop that initiates the malignant phenotype and promotes therapy resistance. Therefore, targeting specific lactylation modifications and their regulatory machinery holds promise for intervention in GC progression and improving therapeutic outcomes at a fundamental level (Table 3). The roles of lactylation in immune cells, including its regulation of tumor-associated macrophages and the establishment of an immunosuppressive network, will be discussed in Section 3.2.

**Table 3:** Mechanisms by Which Protein Lactylation Drives Malignant Phenotypes in GC Cells.

Malignant Phenotype	Key Target/Pathway Affected	Mechanism of Action
Proliferation & Survival	YAP–TEAD Signaling	*AARS1*-mediated lactylation of YAP (K494) stabilizes YAP and promotes its nuclear translocation, activating proliferative genes (*CTGF*, *CYR61*) [26,47,90,91,92].
Invasion & Metastasis	Metabolic–Epigenetic Feedback Loop	GLUT3 overexpression → upregulates *LDHA* → increases lactate production & H3K18la → further epigenetic & metabolic reprogramming [28,29,52].
Therapy Resistance	Chemoresistance	Lactylation-related gene signatures predict differential sensitivity to agents like cisplatin and 5-fluorouracil [29].

### 3.2. The Extrinsic Arm: Lactylation Remodels the Tumor Immune Microenvironment

**Figure 3:**
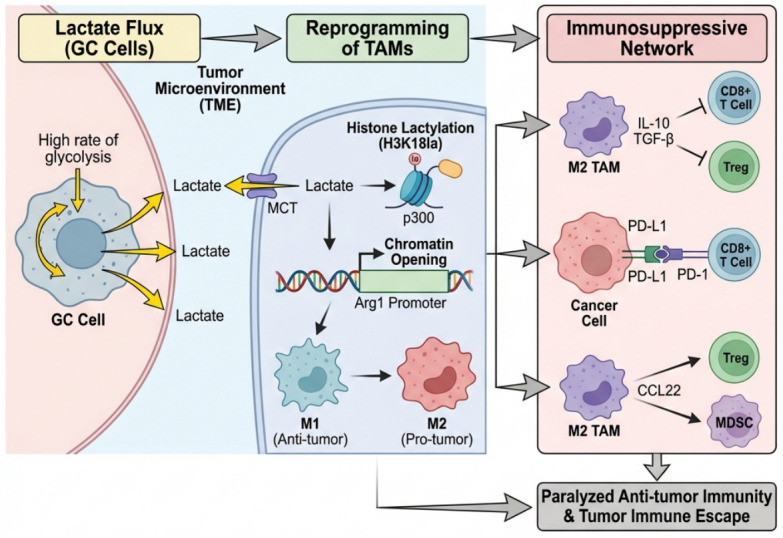
Lactylation-Driven Remodeling of the Immunosuppressive Microenvironment in GC. TAM reprogramming: Lactate enters TAMs via monocarboxylate transporters (MCTs). Immunosuppressive network assembly. All figures were created by the authors using hand-drawing software (including PowerPoint 2023, Adobe Illustrator 2023, and Figdraw online version). No AI-generated images were used.

The tumor immune microenvironment (TIME) of GC is a complex ecosystem comprising cancer cells, stromal cells, and infiltrating immune cells. Recent breakthroughs have revealed that lactate, a metabolic byproduct of tumors, is not merely the end product of glycolysis but also functions as a critical immunoregulatory signaling molecule. A core molecular bridge between cancer cell metabolic reprogramming and systemic immunosuppression is established through the mediation of a novel posttranslational modification known as protein lactylation [11]. Consequently, the local tumor microenvironment becomes enriched with lactate [7,18]. This lactate is subsequently taken up by infiltrating immune cells, particularly macrophages, via monocarboxylate transporters (MCTs). In the cytoplasm, lactate acts as a lactyl donor. Catalyzed by specific “writer” enzymes such as p300, which possesses intrinsic lactyltransferase activity, lactate covalently modifies lysine residues on both histone and nonhistone proteins, thereby altering their function and stability [11,26]. For example, in macrophages, lactate-induced histone H3K18 lactylation (H3K18la) can directly initiate the transcription of immunosuppressive genes such as Arg1, driving macrophage polarization from the antitumor M1 phenotype toward a protumor M2 phenotype that promotes tumor growth, angiogenesis, and immunosuppression [22,23]. This metabolite-driven epigenetic reprogramming ultimately leads to immune cell dysfunction or a shift toward an immunosuppressive state, serving as a key mechanism for tumor immune escape. Among various immune cell types, tumor-associated macrophages (TAMs) represent the most extensively studied and critically important target of this lactate–lactylation signaling axis. The hijacking and functional reshaping of TAMs by lactate via lactylation is a central link in the immunosuppressive shift in the TIME in GC and a core contributor to therapy resistance, providing crucial targets for subsequent therapeutic strategies [22,96] (Figure 3). This section focuses on the role of lactylation in immune cells, with an emphasis on how tumor-derived lactate reprograms TAMs and establishes a multilayered immunosuppressive network.

#### 3.2.1. Key Targets: Lactylation Drives Polarization of Tumor-Associated Macrophages (TAMs) Toward the M2 Phenotype

Lactylation, a lactate-driven posttranslational modification, plays a pivotal role in regulating the polarization of tumor-associated macrophages (TAMs). GC cells produce large amounts of lactate via aerobic glycolysis, which accumulates in the tumor microenvironment (TME) and induces lactylation of histone and nonhistone proteins in macrophages, driving them toward a protumorigenic M2 phenotype [97]. Experimental evidence has shown that lactate treatment of THP-1-derived macrophages significantly upregulates the expression of the M2 marker CD206 (*p* < 0.01), demonstrating that lactate directly activates the M2 polarization program [98].

At the epigenetic level, histone lactylation is a core mechanism regulating the expression of M2-associated genes. Lactylation of histone H3 at lysine 18 (H3K18la) increases chromatin accessibility at the promoters of M2 signature genes (e.g., Arg1), thereby promoting their transcription. For example, in colorectal cancer models, tumor-derived lactate increases H3K18la levels in macrophages, suppresses retinoic acid receptor gamma (RARγ) expression, and promotes M2-like polarization via activation of the *TRAF6*–IL-6–*STAT3* signaling pathway [99]. Similar mechanisms have been validated in hepatocellular carcinoma and glioblastoma, indicating that histone lactylation is a conserved mechanism by which tumor cells reprogram TAMs [100,101].

In addition to histones, lactylation may also modify key signaling transducers or transcription factors within TAMs, cooperatively promoting the M2 polarization program. For example, in GC, collagen type V alpha 2 (*COL5A2*) promotes M2 polarization through lactylation-dependent mechanisms, and its high expression is significantly associated with poor patient prognosis [102]. Additionally, the deubiquitinase *USP37* enhances TAM recruitment by stabilizing Snail1 and is involved in regulating M2 polarization [103].

From a clinicopathological perspective, infiltration of M2-type TAMs in GC tissues is closely associated with disease progression and poor prognosis. M2-like macrophages (*CD163*^+^CD206^+^) constitute the most abundant immune cell population in the GC TME, and their abundance is significantly correlated with advanced tumor stage, lymph node metastasis, and reduced survival [104]. For example, a study of 146 GC patients revealed that the 5-year survival rate was only 35% in patients with high CD206 expression, whereas it was 65% in those with low CD206 expression (*p* < 0.01) [104]. This collective evidence suggests that targeting lactylation and the consequent M2 polarization of TAMs represents a potential strategy for intervening in GC progression and improving patient outcomes.

#### 3.2.2. Establishing an Immunosuppressive Network: Cytokines, Immune Checkpoints, and Recruitment of Suppressive Cells

M2-type tumor-associated macrophages (TAMs), “reprogrammed” by lactylation modifications, serve as central executors in shaping and maintaining the immunosuppressive tumor microenvironment (TME) in GC. They establish a multilayered immunosuppressive network through several synergistic mechanisms, effectively dismantling the host’s antitumor immune response.

First, M2 TAMs directly suppress effector immune cell function by secreting large amounts of immunosuppressive cytokines. They persistently produce high levels of factors such as interleukin-10 (IL-10) and transforming growth factor-beta (TGF-β). IL-10 and TGF-β can directly inhibit the activation, proliferation, and cytotoxic function of CD8^+^ T cells while simultaneously promoting the expansion and stabilization of immunosuppressive regulatory T cells (Tregs). This suppresses effective antitumor immunity at its source.

Second, M2 TAMs and their surrounding TME further “paralyze” the immune system by upregulating immune checkpoint molecules. Research indicates that lactylation can directly or indirectly induce high expression of programmed death-ligand 1 (PD-L1) on both tumor cells and immune cells. One specific mechanism involves cancer-associated fibroblasts (CAFs): CAF-secreted lysyl oxidase (*LOX*) activates the TGFβ signaling pathway, promoting increased lactate production in GC cells. The accumulated lactate, in turn, induces histone H3K18la modification in GC cells, leading to the transcriptional upregulation of PD-L1 expression. This process results in significant functional inhibition of CD8^+^ T cells in the TME (activity is reduced by up to 45%), promoting immune evasion [105]. Additionally, M2 TAMs themselves may highly express checkpoint molecules such as PD-L1, directly engaging PD-1 on T cells to deliver inhibitory signals.

Furthermore, M2 TAMs actively recruit other immunosuppressive cells to amplify the suppressive front. By secreting specific chemokines such as *CCL22* and *CXCL12*, they attract more Tregs and myeloid-derived suppressor cells (MDSCs) to the tumor site [106]. Tregs further suppress effector T-cell function, while MDSCs broadly inhibit both innate and adaptive immunity through mechanisms such as arginine depletion and reactive oxygen species production. This cellular recruitment creates a positive feedback loop, continuously reinforcing the immunosuppressive milieu.

In summary, lactylation-driven M2 TAMs do not function in isolation. Through the triad of mechanisms—“secreting inhibitory factors, upregulating checkpoints, and recruiting suppressive cells”—they interact closely with other components, such as Tregs, MDSCs, and CAFs, collectively weaving a powerful and complex immunosuppressive network. This network effectively neutralizes antitumor immune attacks, providing a sanctuary for GC cell growth, invasion, and metastasis. It represents a key factor contributing to immunotherapy resistance and poor patient prognosis. The comprehensive roles of lactylation in remodeling the GC immune microenvironment are summarized in Table 4.

**Table 4:** Role of Lactylation in Remodeling the GC Immune Microenvironment.

Immune Cell Target	Primary Effect of Lactylation	Key Mechanisms and Downstream Consequences
Tumor-Associated Macrophages (TAMs)	Drives M2 Polarization	Lactate induces histone lactylation (e.g., H3K18la), opening chromatin at M2 gene promoters (e.g., Arg1). Also may lactylate key signaling molecules.
	Establishes Immunosuppressive Network	1. Secretes Inhibitory Cytokines: IL-10, TGF-β to suppress CD8^+^ T cells and promote Tregs. 2. Upregulates Checkpoints: Induces PD-L1 expression. 3. Recruits Suppressive Cells: attracts Tregs and MDSCs via chemokines (*CCL22*, *CXCL12*).
Other Immune Cells (Potential)	Modulates Function	1. Dendritic Cells (DCs): May impair maturation and antigen presentation. 2. T Cells: May directly suppress CD8^+^ T cell function or stabilize Tregs. 3. Neutrophils (TANs): May drive polarization toward pro-tumor N2 phenotype.

### 3.3. Integration: The Self-Reinforcing Metabolic–Epigenetic–Immunological Circuit

#### 3.3.1. The Circuit Architecture

The self-reinforcing circuit originates from the exceptionally active Warburg effect in GC cells, driven by oncogenic alterations, hypoxia, and factors such as H. pylori infection [5,6,7,8,16,17,18,19]. This metabolic reprogramming leads to massive lactate production and secretion into the tumor microenvironment [7,9].

Within cancer cells, lactate serves as a direct substrate for lactylation modifications. Key “writer” enzymes such as *AARS1* and p300 catalyze the lactylation of critical regulators including YAP and histones [26,47,52]. This modification activates oncogenic signaling pathways (e.g., YAP–TEAD), promotes proliferation and invasion, and—critically—further upregulates glycolytic enzymes such as *LDHA*, creating a positive feedback loop that amplifies lactate production [28,29,52]. Thus, an intrinsic loop is established: the Warburg effect fuels lactylation, which in turn reinforces glycolysis, generating even more lactate.

Simultaneously, lactate exported from cancer cells enters immune cells, particularly tumor-associated macrophages (TAMs), via monocarboxylatetransporters (MCTs) [22,23]. These M2 TAMs execute a multifaceted immunosuppressive program: they secrete IL-10 and TGF-β to suppress CD8^+^ T cells, upregulate PD-L1 to deliver inhibitory signals, and recruit Tregs and MDSCs via chemokines [96,104,105,106]. The resulting immunosuppression allows tumors to grow and metastasize, which in turn increases metabolic demand and further amplifies the Warburg effect. This extrinsic loop can be summarized as follows: tumor-derived lactate drives TAM lactylation and M2 polarization, leading to immunosuppression that promotes tumor growth, which further enhances lactate production.

The intrinsic and extrinsic loops are not independent; they are tightly coupled through two synergistic mechanisms. First, lactate serves as the common fuel for both loops. Lactate produced by cancer cells via the Warburg effect is the very same molecule that enters TAMs and drives their lactylation-dependent reprogramming. Consequently, any amplification of glycolytic flux within cancer cells directly fuels immunosuppression. Second, tumor growth acts as a self-amplifier. Enhanced tumor growth, driven by both intrinsic malignant progression and extrinsic immune evasion, increases overall metabolic demand, further activating the Warburg effect and producing more lactate.

#### 3.3.2. Functional Consequences of the Self-Reinforcing Circuit

This self-reinforcing circuit has profound implications for GC progression and therapy. First, it ensures sustained malignancy: once established, the malignant state becomes self-perpetuating; even if one component is temporarily inhibited, the circuit can reactivate through alternative nodes. Second, the circuit explains therapy resistance: targeting cancer cell metabolism alone may be insufficient because the immunosuppressive microenvironment remains intact and continues to support tumor growth; conversely, targeting immune checkpoints alone is limited by sustained metabolic reprogramming that fuels immunosuppression [26,27,95,96,105]. Third, the circuit reveals metabolic–immune coupling as a central principle: metabolic reprogramming and immune evasion are not separate phenomena but are mechanistically coupled through lactylation. This positions lactylation as a central hub where these two hallmarks of cancer converge.

**Figure 4:**
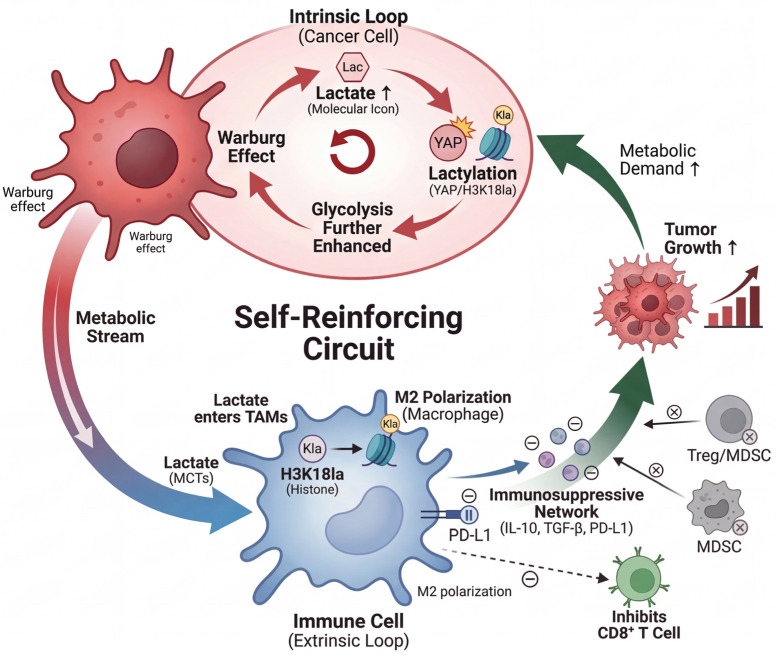
The Self-Reinforcing Metabolic–Epigenetic–Immunological Circuit in GC. This schematic illustrates how protein lactylation integrates intrinsic and extrinsic arms into a self-reinforcing circuit. Intrinsic Arm: Warburg effect in GC cells leads to lactate production. Lactate fuels lactylation of YAP and histones via “writers” (*AARS1*, p300), activating oncogenic pathways that further amplify glycolysis. Extrinsic Arm: Lactate exported from cancer cells enters TAMs, driving histone lactylation and M2 polarization. M2 TAMs establish immunosuppression via cytokines (IL-10, TGF-β), PD-L1, and recruitment of Tregs/MDSCs, which suppress CD8^+^ T cells and promote tumor growth. Tumor growth further amplifies the Warburg effect, closing the loop. Lactylation serves as the central hub coupling these two arms. All figures were created by the authors using hand-drawing software (including PowerPoint 2023, Adobe Illustrator 2023, and Figdraw online version). No AI-generated images were used.

Recognizing lactylation as the hub of a self-reinforcing circuit suggests that effective therapeutic strategies should aim to disrupt the circuit at multiple levels. At the source (the Warburg effect), inhibiting key glycolytic enzymes such as *LDHA* may reduce lactate availability, dampening both intrinsic and extrinsic loops [52,54]. Within the intrinsic loop, targeting lactylation “writers” such as *AARS1* or p300 can directly block lactylation-driven malignant phenotypes in cancer cells [26,45,47]. Within the extrinsic loop, activating delactylases such as *SIRT1* or blocking lactylation-driven M2 polarization can restore antitumor immunity [27,69,96]. Importantly, because the two loops are coupled, combination strategies that simultaneously target multiple nodes may be more effective than any single approach. For example, combining a lactylation inhibitor with an immune checkpoint inhibitor could disrupt the circuit’s self-reinforcing nature and overcome resistance [96,105,107] (Figure 4).

### 3.4. Physiological Roles of Lactylation: Insights from Tissue Repair, Gut Infection, and Immune Surveillance

The preceding sections have focused on the pathological roles of lactylation in GC. However, as the reviewer insightfully noted, understanding the physiological functions of lactylation is essential for contextualizing its disease-associated dysregulation. Emerging evidence indicates that lactylation operates on a continuum from homeostasis to pathology, playing context-dependent roles in normal tissue repair, host defense against gut infection, and the regulation of immune surveillance under steady-state conditions [108]. Lactylation in normal tissue repair and wound healing: Lactylation was first identified in tissue-repairing macrophages, where it regulates gene expression during the resolution phase of inflammation [108]. Macrophage-derived lactate acts as a mediator of fibroblast phenotypic remodeling via MCT1-mediated histone H3 lysine 23 lactylation (H3K23la), promoting transcriptional activation of repair-associated genes [109]. Within macrophages themselves, lactylation influences inflammatory signaling and immune cell polarization, providing a conceptual link across tissue repair processes [110].

Lactylation in gut infection and intestinal inflammation: The intestinal tract represents a particularly relevant context for understanding lactylation biology, given the convergence of host metabolism, immune function, and microbiota-derived signals [111]. In the context of gut infection and intestinal inflammation, lactylation drives macrophage phenotypic conversion, mediates gut microbiota–host interactions, regulates fibrosis progression, and modulates intestinal inflammation and tissue repair [112]. This finding illustrates a tripartite interaction among gut microbiota, host lactylation machinery, and therapeutic response. Beyond bacterial infections, lactylation also plays regulatory roles in viral gastrointestinal infections [113]. Importantly, lactylation exhibits dual regulatory roles in macrophage-mediated immune responses: it fosters anti-inflammatory and reparative phenotypes under certain conditions, yet may expedite tumor progression and induce immunosuppression in gastrointestinal malignancies [112].

Lactylation in immune surveillance under normal conditions: Lactylation functions as a rheostat that fine-tunes immune surveillance in the steady state. Regarding innate immune surveillance, AARS1 (alanyl-tRNA synthetase)—discussed extensively as a lactyltransferase in GC pathology—also functions under physiological conditions as a sensor of L-lactate that conveys repression of innate immune surveillance [114]. Elevated L-lactate levels are inversely correlated with cGAS-mediated immune responses, and *AARS1*-mediated lactylation directly represses innate immune activation, suggesting that lactylation helps establish a baseline threshold to prevent unwarranted inflammatory responses [114]. In macrophages—key sentinels of immune surveillance—histone lactylation regulates gene transcription during activation, and the balance between lactylation and delactylation (mediated by “erasers” such as HDAC1-3 and SIRT1/2) determines the overall immune tone [85]. Constitutive, low-level lactylation in resting immune cells suggests a homeostatic “set point” that can be modulated in response to metabolic cues. Regarding adaptive immune surveillance, while most studies have focused on pathological high-lactate environments, physiological lactate fluctuations may fine-tune CD8^+^ T cell and Treg function. The existence of dedicated “writers” (p300/CBP, *AARS1*, GCN5, PCAF) and “erasers” (*SIRT1*, *SIRT2*, HDAC1-3) that operate under basal metabolic conditions strongly supports the notion that lactylation is a constitutive regulatory mechanism, not merely a pathological response to metabolic stress [115].

Implications for the pathological framework in GC: A unifying theme emerging from these physiological studies is the context-dependent nature of lactylation biology. Lactylation exhibits a “paradoxical modulation” role, wherein it can be either protective or pathogenic depending on the cellular context, duration of activation, and metabolic environment. Lactylation promotes anti-inflammatory and reparative phenotypes in normal tissue repair and resolution of inflammation, yet when co-opted in the tumor microenvironment, it drives immunosuppression and malignant progression. First, complete ablation of lactylation may have unintended consequences for tissue homeostasis and immune function. Second, context-specific therapeutic targeting—for example, selectively inhibiting pathological lactylation driven by oncogenic “writers” such as *AARS1* while preserving physiological lactylation mediated by distinct enzymatic complexes—is desirable. Third, the efficacy of lactylation-targeting agents may depend on baseline lactylation status, which could vary across patients and disease stages.

### 3.5. Dietary and Nutritional Modulation of Protein Lactylation: Implications for Therapy

Emerging evidence suggests that dietary factors may influence lactylation by modulating systemic lactate availability, though direct evidence in GC remains limited.

High-fat diets have been shown to cooperate with oncogenic drivers to induce glycolysis and lactate accumulation in tumors, potentially amplifying lactylation-driven signaling [116]. High-sugar intake promotes lactylation of non-histone proteins (e.g., *RCC2*) in breast cancer models [117], and epidemiological studies link processed meat and salt-preserved foods to GC risk [118]. However, direct evidence connecting these dietary patterns to protein lactylation in GC is lacking.

Gut microbiota may influence host lactylation. Meta-analyses have found Lactobacillus enrichment in GC tissues [119], and in colorectal cancer, Roseburia intestinalis intervention reduced cellular lactylation [120]. Whether similar effects occur in GC remains unknown.

Ketogenic diets restrict glucose availability and may reduce lactate production from the Warburg effect [121]. Caloric restriction has been shown to reprogram CD8^+^ T cell metabolism and enhance anti-tumor immunity [122]. These strategies could theoretically lower substrate availability for lactylation, but clinical feasibility in GC patients is uncertain, particularly given risks of cachexia and malnutrition.

Almost all evidence for diet–lactylation links comes from non-GC models. Distinguishing dietary lactate from endogenously produced lactate is critical, as gut-derived lactate may not directly reach tumor cells. Future research requires GC-specific preclinical models and prospective clinical studies before any dietary recommendations can be made.

## 4. Clinical Translation Potential and Challenges

### 4.1. As a Novel Biomarker: Potential from Bench to Bedside Detection

Protein lactylation, which serves as a direct readout at the intersection of metabolic reprogramming and epigenetic regulation, holds significant theoretical promise as an ideal biomarker. Its levels dynamically reflect tumor metabolic activity, malignancy, and the immune microenvironment status, forming the basis for its potential application in the diagnosis, prognosis, and treatment response prediction of GC.

With respect to prognostic and diagnostic value, specific lactylation markers in tissue samples have shown strong correlations with clinical outcomes. For example, high levels of histone H3K18la are significantly associated with poor prognosis in GC patients, with a 5-year survival rate of only 28% in high-expression patients compared with 62% in low-expression patients [34]. Nonhistone lactylation also has prognostic value; for example, lactylation of nucleolin (*NCL*) at Lys477 is linked to increased metastasis rates, detected in 68% of metastatic cases versus 24% of nonmetastatic cases [35]. These findings position specific lactylation marks as powerful tissue-based prognostic indicators.

Liquid biopsy offers a minimally invasive approach for detecting lactylation markers. Studies have demonstrated the feasibility of detecting lactylated proteins in plasma via techniques such as liquid chromatography–tandem mass spectrometry (LC–MS/MS), which achieve a sensitivity of 85% and specificity of 78% in GC patient plasma [34]. Furthermore, noninvasive prediction models based on lactylation-related gene expression are under development. A 7-gene immune microenvironment-related prognostic signature (IMPS), which includes genes such as *CTLA4*, achieved an area under the curve (AUC) of 0.802 for predicting 5-year survival in patients with GC, demonstrating good predictive performance [36].

Lactylation signatures have potential for the prediction of therapeutic response. A 13-gene signature linking hypoxia, glycolysis, and lactylation (HGLRGs) not only predicts patient survival (3-year AUC = 0.764) but also distinguishes sensitivity to chemotherapeutic agents such as 5-fluorouracil and oxaliplatin, with a 2.1-fold reduction in predicted IC50 values for high-risk patients [37]. Mechanistically, high lactylation levels are often associated with a more immunosuppressive microenvironment (e.g., via induction of PD-L1 expression), potentially influencing patient response to immune checkpoint inhibitors (e.g., anti-PD-1/PD-L1 therapy), making it a candidate biomarker for predicting immunotherapy efficacy.

The detection technologies underpinning this research are continually advancing. High-sensitivity mass spectrometry remains the gold standard for discovering and quantifying lactylation sites [37]. However, translating these advanced techniques into routine clinical practice faces challenges, including the standardization of protocols, cost reduction, further improvements in sensitivity and specificity, and validation in large-scale prospective cohorts. Overcoming these hurdles is crucial for realizing the clinical application of protein lactylation as a biomarker in GC. The key clinical translation potential and challenges of targeting lactylation in GC are summarized in Table 5.

**Table 5:** Clinical Translation Potential and Challenges of Targeting Lactylation in GC.

Aspect	Specific Approach/Target	Major Challenges & Future Needs
Biomarker Potential	Tissue-based: H3K18la levels, *NCL* lactylation (Lys477) [84].	Requires validation in large-scale, prospective cohorts; invasive nature of tissue sampling.
	Liquid Biopsy: Plasma lactylated proteins, lactylation-related gene signatures (e.g., HGLRG, IMPS).	High cost and complexity of advanced techniques (e.g., mass spectrometry); need for standardized, sensitive clinical assays.
Therapeutic Strategies	Inhibit Writers: *AARS1*, p300/CBP inhibitors [26].	Need for inhibitors selective for lactyltransferase over acetyltransferase activity; metabolic plasticity may lead to resistance.
	Activate Erasers: *SIRT1*/*SIRT2* activators.	Complexity of enzyme multifunctionality (e.g., HDAC inhibitors may block both deacetylation and delactylation).
	Combination Therapy: Lactylation pathway inhibitors + Immune Checkpoint Inhibitors/Chemotherapy.	Optimal combinations, sequencing, and patient selection criteria are undefined.
Future Tools & Directions	Specific Reagents: High-affinity antibodies, selective chemical probes, nano-based detection platforms.	Current lack of tools that reliably distinguish lactylation from similar PTMs (e.g., acetylation).
	Panoramic Mapping: Single-cell lactylomics, spatial lactylomics.	Technically challenging and requires sophisticated data analysis.

### 4.2. Novel Therapeutic Strategies Targeting the Lactylation Pathway

On the basis of the previously elucidated core role of lactylation in GC metabolic reprogramming, immune microenvironment remodeling, and tumor progression, targeting the lactylation pathway has emerged as a highly promising novel therapeutic paradigm. Current strategies focus primarily on three key aspects: inhibiting the generation of lactylation modifications (targeting “writers”), promoting their removal (targeting “erasers”), and combining lactylation modulators with existing therapies to increase antitumor efficacy.

#### 4.2.1. Inhibiting the “Writers”: Blocking the Formation of Lactylation

Directly targeting lactyltransferases, or “writers,” represents a logical and promising strategy to suppress lactylation-driven oncogenic signaling in GC.

Alanyl-tRNA synthetase 1 (*AARS1*), a classical enzyme in protein synthesis, has recently been identified as a bona fide lactyltransferase with a moonlighting function in cancer epigenetics. In GC, *AARS1* is frequently upregulated and translocates to the nucleus, where it directly utilizes lactate and ATP to lactylate YAP at specific lysine residues (e.g., Lys494) [26]. This modification enhances YAP nuclear translocation and transcriptional activity, activating the YAP-TEAD signaling axis and promoting tumor proliferation and survival [26]. Preclinical evidence strongly supports AARS1 as a therapeutic target: knockdown of *AARS1* in GC models significantly reduces YAP lactylation levels (by 72%) and suppresses tumor growth (by 55–58%) in xenograft studies [26,47]. These findings highlight the potential of developing specific inhibitors, such as small molecules or small interfering RNA (siRNA)-based therapeutics, to disrupt *AARS1*-mediated lactylation and its downstream oncogenic effects.

The histone acetyltransferases p300 and CBP also exhibit lactyltransferase activity and are crucial for histone lactylation, particularly at sites such as H3K18la, which is associated with poor prognosis in GC [45]. Inhibiting p300/CBP provides a means to broadly reduce lactylation-mediated epigenetic reprogramming. In preclinical studies, the small-molecule p300 inhibitor C646 effectively reduced global H3K18la levels and inhibited GC cell proliferation [45]. The translational potential of this approach is being actively explored in early-phase clinical trials. For example, trials such as NCT04880062 and NCT05904106 are evaluating p300/CBP inhibitors in patients with advanced solid tumors, including GC, and assessing their safety, tolerability, and preliminary antitumor efficacy [123].

Beyond *AARS1* and p300/CBP, other enzymes with lactyltransferase activity or those that critically regulate lactate availability (e.g., *LDHA*) are also considered indirect “writers.” Inhibiting *LDHA*, for example, reduces the substrate (lactate) for lactylation, thereby suppressing this modification and its downstream effects [27]. However, direct inhibition of catalytic “writers”, such as *AARS1* and p300/CBP, offers a more precise strategy to block lactylation at its source.

In summary, the inhibition of lactylation “writers,” particularly *AARS1* and p300/CBP, holds significant therapeutic promise. Robust preclinical data demonstrate that targeting these enzymes effectively suppresses lactylation, disrupts key oncogenic pathways, and inhibits GC progression. Ongoing clinical trials will determine the feasibility and efficacy of this strategy in patients, potentially establishing lactyltransferase inhibitors as a novel component of precision therapy for GC.

#### 4.2.2. Modulating the “Erasers”: Manipulating the Dynamic Balance of Lactylation

Targeting delactylases, or “erasers,” represents a strategic approach to reverse lactylation-driven oncogenic signaling by promoting the removal of lactyl groups from proteins. The core principle involves enhancing the activity of endogenous delactylases (e.g., HDAC1-3, *SIRT1*, and *SIRT2*) or inhibiting their negative regulators, thereby accelerating the clearance of lactylation modifications and restoring normal epigenetic and metabolic homeostasis in GC cells.

Sirtuins, particularly *SIRT1*, have been identified as key histone delactylases in GC. *SIRT1* directly removes lactyl groups from histone H3 at lysine 18 (H3K18la), and its loss or inhibition leads to H3K18la accumulation, which in turn activates a positive feedback loop involving the lncRNA *H19*, enhances glycolysis, and further enhances lactylation, driving tumor progression [27]. Conversely, activating or restoring *SIRT1* activity could disrupt this cycle and suppress oncogenic transcription. Similarly, *SIRT2* has been shown to delactylate nonhistone proteins, such as *METTL16*, thereby influencing downstream pathways such as the cuproptosis pathway [69]. Therefore, developing specific small-molecule activators (e.g., *SIRT1* activators such as SRT2104) to increase delactylase activity offers a direct and precise strategy to reduce global lactylation levels and inhibit GC growth.

#### 4.2.3. Combination Therapy: Synergizing with Immune Checkpoint Inhibitors to Reverse Immunosuppression

Although the strategies described above each demonstrate therapeutic potential, they differ significantly in target characteristics, selectivity, applicable disease stages, and therapeutic advantages. Clarifying these differences is essential for designing rational combination regimens and achieving precision therapy. A cross-comparison of major targets is summarized in Table 6.

It is important to note that HDAC inhibitors exhibit a complex “double-edged sword” effect in the regulation of lactylation. Studies have shown that *HDAC1*, *HDAC2*, and *HDAC3* all possess delactylase activity and are capable of hydrolyzing lactyl groups on histones [72,73,85]. Therefore, theoretically, classical HDAC inhibitors (such as vorinostat) block delactylase activity while inhibiting HDAC deacetylase function, potentially leading to aberrantly elevated intracellular lactylation levels—a finding that appears contradictory to their expected antitumor effects. However, preclinical studies have demonstrated that vorinostat still suppresses tumor growth in GC models (by approximately 40%) [85], suggesting that its broad-spectrum epigenetic effects (e.g., influencing multiple histone modifications and transcriptional programs) may dominate the overall outcome and offset any adverse consequences associated with increased lactylation. This paradox underscores the need for more precise regulation of lactylation homeostasis in future research: on one hand, selective HDAC isoform inhibitors should be developed to distinguish between deacetylase and delactylase functions; on the other hand, combining HDAC inhibitors with lactylation “writer” inhibitors (e.g., p300 inhibitors) may help circumvent the risks of monotherapy. The potential advantages and limitations of this strategy are systematically compared in Table 4.

**Figure 5:**
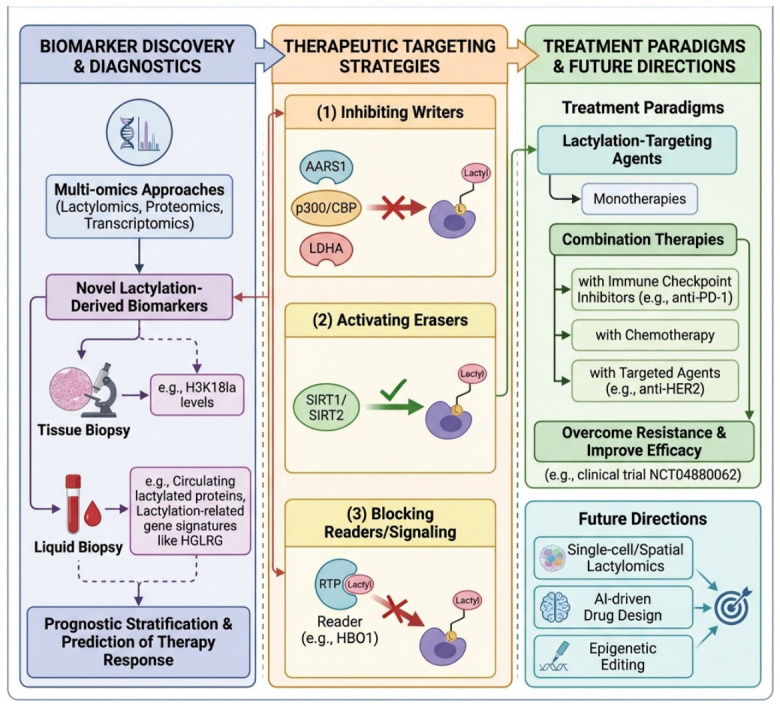
Roadmap for Clinical Translation: Targeting the Lactylation Pathway in GC. This roadmap outlines the potential clinical translation of lactylation research in GC, spanning from diagnosis to therapy. Diagnostic Biomarker Development: Lactylation-derived markers discovered via multiomics can be applied in tissue or liquid biopsies for prognostic stratification and treatment response prediction. Therapeutic Targeting Strategies: Three primary intervention strategies are inhibition of lactyltransferase “Writers”; activation of delactylase “Erasers”; and blockade of “Reader” protein function. Treatment Integration & Future Directions: Lactylation-targeting agents hold promise both as monotherapies and, more effectively, in rational combination strategies with existing standards of care. All figures were created by the authors using hand-drawing software (including PowerPoint 2023, Adobe Illustrator 2023, and Figdraw online version). No AI-generated images were used.

In summary, the inhibition of lactylation “writers,” particularly *AARS1* and p300/CBP, holds significant therapeutic promise, with robust preclinical data demonstrating effective suppression of lactylation and disruption of key oncogenic pathways. Modulating “erasers” represents a promising but nuanced strategy; moving beyond nonspecific HDAC inhibitors, future efforts should focus on developing selective delactylase activators (e.g., for *SIRT1/SIRT2*) or isoform-specific HDAC modulators. Such precision tools would allow for the targeted restoration of lactylation homeostasis. Critically, the success of these approaches will depend on rational combination strategies that simultaneously disrupt multiple nodes of the self-reinforcing metabolic–epigenetic–immunological circuit. Ongoing and future clinical trials will determine the feasibility and efficacy of these strategies in patients, potentially establishing lactylation-targeting agents as a novel component of precision therapy for GC (Figure 5).

## 5. Future Perspectives

Despite groundbreaking insights, lactylation research in GC remains nascent. To unlock its full biological and therapeutic potential, future efforts must focus on three interconnected frontiers: panoramic mapping, reader elucidation, and clinical translation.

### 5.1. Mapping the Panoramic and Dynamic Landscape of Lactylation in GC

A comprehensive understanding requires moving beyond bulk analyses to chart the heterogeneous and dynamic distribution of lactylation across the tumor ecosystem. Leveraging emerging technologies—single-cell lactylomics (integrating scRNA-seq with sensitive mass spectrometry), spatial transcriptomics/proteomics, and artificial intelligence—will be pivotal.

Key research goals include: resolving cellular heterogeneity by determining the specific distribution and function of lactylation across malignant subclones, immune cells (TAMs, T cells), and cancer-associated fibroblasts (CAFs); mapping spatiotemporal dynamics by tracing lactylation marks during tumor initiation, progression, therapy response, and resistance development; and investigating subtype specificity by comparing lactylation profiles across GC molecular subtypes (e.g., EBV-positive, MSI-H, genomically stable) to identify subtype-specific vulnerabilities and biomarkers. These efforts will provide an unprecedented systems-level view of GC heterogeneity, drug resistance mechanisms, and novel therapeutic targets [37].

### 5.2. Deciphering the “Readers” and Precise Regulatory Network of Lactylation

While knowledge of lactylation “writers” (*AARS1*, p300) and “erasers” (*SIRT1*, HDACs) is increasing, identifying the specific “reader” proteins that recognize lactylation marks and mediate downstream effects remains a critical gap. Proteomic screening (e.g., with lactylated peptide baits) and structural biology can help validate reader domains (e.g., bromodomains, YEATS domains) that bind distinct lactylation sites such as H3K18la, with initial studies like *TRIM33*’s bromodomain binding to H3K18la paving the way [81]. Equally important is deconstructing how writers, erasers, and readers form a dynamic regulatory circuit and crosstalk with other PTMs (e.g., acetylation, methylation). Deciphering readers is essential for understanding lactylation-driven gene regulation and provides a theoretical foundation for drugs that interfere with signal readout.

Despite progress in identifying lactylation readers, major challenges remain. Currently, the number of validated readers in GC is limited, and the roles of many potential recognition proteins (e.g., those containing YEATS domains) remain unexplored [126]. Reader functions are highly context-dependent, and the same protein may exert opposing effects in different GC subtypes or cellular environments. A critical bottleneck is the lack of highly selective probes or tools that can distinguish lactylation from structurally similar modifications such as acetylation, which severely limits mechanistic studies [32,33]. Moreover, the molecular mechanisms governing selective recognition between lactylation and acetylation are still unclear; whether dedicated readers exist that specifically recognize Kla but not Kac, and what their structural basis might be, await future investigation. To address these challenges, future efforts should focus on: systematically screening for GC-specific lactylation readers using advanced proteomic approaches; elucidating recognition interfaces at atomic resolution through structural biology; developing small molecules or peptide inhibitors that disrupt reader–lactylation mark interactions; and exploring the clinical potential of readers as prognostic biomarkers or therapeutic targets [31,127]. With the continued advancement of multiomics integration and single-cell technologies, the reader network in GC is expected to be further unraveled, paving the way for combined metabolic–epigenetic therapies.

### 5.3. Toward Clinical Translation: From Biomarkers to Targeted Therapies

Translating basic discoveries into clinical applications requires pragmatic pathways. Promising lactylation biomarkers—circulating lactylated proteins, tissue H3K18la levels, or gene signatures like HGLRG [81]—must be validated in large-scale, prospective, multicenter studies to establish their utility for early detection, prognostic stratification, and prediction of immunotherapy response.

In terms of innovative therapeutic exploration, four directions merit attention. First, drug development should focus on high-specificity agents: small molecules that selectively inhibit p300/CBP lactyltransferase over acetyltransferase activity [81]; compounds modulating specific delactylases (e.g., *SIRT1* activators); and inhibitors blocking critical lactylation readers. Second, rational combination strategies should be tested based on mechanistic insights—e.g., lactylation pathway inhibitors with immune checkpoint inhibitors, chemotherapy, or targeted therapies (e.g., anti-HER2)—to reverse immune suppression and overcome resistance. Early-phase trials combining p300 inhibitors with anti-PD-1 therapy (NCT04880062) exemplify this effort [107]. Third, novel modalities such as cell-based therapies (e.g., CAR-T cells recognizing lactylation-induced neoantigens) or epigenetic editing targeting specific lactylation sites should be explored. Fourth, interdisciplinary integration across metabolic biology, epigenetics, immunology, medicinal chemistry, and clinical oncology will be essential.

In summary, the future of lactylation research in GC lies at the intersection of technological innovation, mechanistic rigor, and clinical pragmatism. By embracing complexity, deciphering molecular readers, and forging robust translational pathways, the field is poised to deliver transformative advances in diagnosing, treating, and understanding this devastating disease.

## 6. Conclusions

The journey of lactate from a dismissed waste product to a central signaling molecule mirrors a broader shift in cancer biology: the realization that metabolism, epigenetics, and immunity are not separate domains but components of an integrated regulatory network. In GC, protein lactylation stands at the intersection of these domains, serving as the molecular translator that converts metabolic flux into epigenetic instructions and cellular behavior [11,12,13,14,15,24,25].

We have proposed that lactylation functions as a central hub with two synergistic arms. Intracellularly, it directly fuels malignant phenotypes—proliferation, invasion, metastasis, and therapy resistance—by modifying key regulators such as YAP, metabolic enzymes, and RNA-binding proteins [26,28,29,35,47,51,90,93]. Extracellularly, it remodels the tumor immune microenvironment by reprogramming tumor-associated macrophages toward an immunosuppressive M2 phenotype [22,23,96,97,98,99,100,101,102,103,104], upregulating immune checkpoints such as PD-L1 [96,105], and establishing a network of inhibitory signals that silence antitumor immunity [106,128,129,130]. These two arms do not operate in isolation; they converge into a self-reinforcing metabolic–epigenetic–immunological circuit that perpetuates the Warburg effect and locks the tumor–immune system into a progressively malignant state [7,26,27,28,29,130].

This reframing carries three implications. First, lactylation is an actionable vulnerability—a convergence point for disrupting multiple oncogenic processes [48,51,95]. Second, effective therapy must target both arms; targeting cancer cells alone is insufficient if the immunosuppressive microenvironment persists [96,105,131]. Third, context-specific approaches are needed, as lactylation effects depend on cell type, disease stage, and metabolic landscape [37,132].

Major challenges remain. Most evidence is correlative, with scarce causal proof [132]. High-fidelity tools—lactylation-specific antibodies, selective inhibitors, and site-specific genetic models—are lacking [32,33]. The heterogeneity and dynamic regulation of lactylation remain largely unexplored [34,37].

Three breakthroughs are essential for clinical translation: (i) spatially resolved mapping of the lactylation landscape using single-cell and spatial omics to define functionally consequential events [34,37]; (ii) transition from correlation to causation via site-specific lactylation mutants and chemical biology tools [74,75,105,106]; and (iii) context-aware therapeutic strategies targeting oncogenic circuits (e.g., *AARS1*–YAP, H3K18la–M2) guided by biomarker-enriched trials, rather than global lactylation inhibition [22,23,26,27,36,37,47,96,107].

In conclusion, protein lactylation represents far more than a novel posttranslational modification. In GC, it is the molecular bridge that links metabolic reprogramming to malignant progression and immune evasion [11,12,24,25], the amplifier that sustains the Warburg effect [7,26,27], and the unifying principle that integrates three hallmarks of cancer into a coherent, targetable framework [14,15,48]. By recognizing lactylation as a central hub rather than a peripheral detail, we open the door to a new class of therapeutic strategies—ones that disrupt the self-reinforcing circuit at its core, offering the potential to simultaneously curb cancer cell malignancy and restore antitumor immunity. The path forward demands rigorous mechanistic inquiry, innovative tool development, and a willingness to challenge the fragmented view of cancer biology. If successful, lactylation-based precision medicine may fundamentally change how we understand and treat GC.

## Figures and Tables

**Table 1:** Summary of Lactylation “Reader” Recognition Characteristics.

Feature Dimension	Commonality	Specificity
Chemical Basis	Hydrogen bonding and hydrophobic interactions	Hydroxyl group of lactyl moiety can form additional hydrogen bonds
Domain Type	Bromodomain, SH2 domain, etc.	YEATS domains and others await validation
Binding Affinity	Comparable affinity for Kla and Kac (e.g., HBO1)	Specific domain mutants may selectively recognize Kla
Functional Output	Transcriptional activation, protein stabilization, signal transduction	Often associated with metabolic stress and immune remodeling

**Table 2:** Summary of Key Enzymes/Proteins Involved in Protein Lactylation in GC.

Category	Enzyme/Protein	Key Function/Description
Writers	p300/CBP [85]	Catalyzes histone lactylation (e.g., H3K18la) implicated in GC progression inhibitor C646 reduces GC cell proliferation.
	*AARS1* [26]	Directly lactylates YAP (e.g., K494) using lactate ATP activating YAP-TEAD signaling to promote proliferation; overexpression correlates with poor prognosis.
	*LDHA* [52]	Upregulated in GC; correlates with increased global lactylation and poor prognosis; part of a metabolic-epigenetic feedback loop.
	GCN5/PCAF [86]	Contribute to lactylation in inflammatory/hypoxic tumor microenvironments.
	*HNRNPA2B1* [87]	Undergoes auto-lactylation in lactate-rich conditions, stabilizing oncogenic mRNAs in GC cells.
Erasers	*SIRT1* [27]	Low expression leads to H3K18la accumulation drives a pro-tumorigenic feedback loop involving lncRNA *H19* and glycolysis correlates with poor prognosis
	*SIRT2* [69]	Delactylates *METTL16* (K229) inhibiting its m6A activity and influencing cuproptosis in GC
	*HDAC1/HDAC3* [85]	Likely involved in balancing the lactylation network in GC
Readers	HBO1 [79]	Recruited to target gene promoters promoting transcription of cell cycle-related genes driving GC proliferation.
	*PFKM* [84]	Interacts with H3K18la to promote transcriptional activation of the *CNTN1* gene enhancing GC cell invasion.
	*EGFR/SRC* [82]	Bind lactylated *GPX4* enhancing its stability and inhibiting ferroptosis, promoting diabetes-associated GC.
	*USP7* [88]	Interacts with lactylated proteins; its expression positively correlates with lactylation levels in GC tissues and indicates poor prognosis.

**Table 6:** Differentiated Comparison of Major Lactylation-Targeting Strategies in GC.

Feature Dimension	*AARS1* [26,124] Inhibitors	p300/CBP Inhibitors [125]	*SIRT1/SIRT2* Activators [27]	HDAC Inhibitors (Nonspecific) [85]
Target Type	“Writer” (lactyltransferase)	“Writer” (dual acetyl/lactyltransferase)	“Eraser” (delactylase)	“Eraser” (HDAC1-3 possess delactylase activity)
Mechanism of Action	Directly blocks lactylation of key proteins (e.g., YAP)	Broadly inhibits histone and nonhistone lactylation	Promotes hydrolysis of lactyl groups, reversing lactylation	Inhibits dual deacetylase/delactylase functions
Selectivity	High (*AARS1* overexpressed in GC with relative tumor specificity)	Low (simultaneously inhibits acetylation and lactylation)	Moderate (requires distinction among SIRT family members)	Very low (affects multiple HDAC substrates)
Primary Substrates	Nonhistone proteins such as YAP (K494)	Histone lactylation such as H3K18la	H3K18la, *METTL16*, etc.	Broad-spectrum histones and transcription factors
Advantages	High specificity; targets defined oncogenic pathway (YAP-TEAD)	Broad-spectrum antitumor effects; existing clinical trial infrastructure	Restores endogenous regulatory homeostasis; avoids complete ablation of lactylation	Some agents already approved; clear clinical translation pathway
Limitations	*AARS1* expressed in normal tissues; long-term inhibition may affect protein synthesis	On-target toxicity (cardiac, hepatic); difficulty separating acetyl- from lactyl-transferase inhibition	Selectivity and bioavailability of activators require optimization	May paradoxically increase lactylation; complex toxicity profile
Applicable Stage	Early/locally advanced GC; YAP-signaling-high subtypes	Advanced GC; combination therapy settings	Early intervention; maintenance therapy; prevention of acquired resistance	Approved for hematologic malignancies; limited efficacy in solid tumors
Development Stage	Preclinical (small molecule screening; initial PROTAC exploration)	Phase I-II clinical (CCS1477, CPI-1612, etc.)	Preclinical to early clinical (SRT2104, etc.)	Clinically approved (exploratory in solid tumors)

## Data Availability

No new data were created or analyzed in this study. Data sharing is not applicable to this article.

## Abbreviations

The following abbreviations are used in this manuseript:

GC	GC
TME	Tumor microenvironment
Kla	protein lactylation
H. pylori	Helicobacter pylori
*LDHA*	Lactate dehydrogenase A
PKM2	Pyruvate kinase M2
H3K18la	Histone H3 Lysine 18 Lactylation
*AARS1*	Alanyl-tRNA Synthetase 1
YAP	Yes-associated protein
TEAD	Transcriptional Enhanced Associate Domain
*SIRT1/2/3/5*	Sirtuin 1/2/3/5
*HDAC1/3*	Histone Deacetylase 1/3
*METTL16*	Methyltransferase-like 16
m6A	N6-methyladenosine
*FDX1*	Ferredoxin 1
*EGFR*	Epidermal growth factor receptor
*SRC*	SRC Proto-Oncogene, Nonreceptor Tyrosine Kinase
*GPX4*	Glutathione Peroxidase 4
*USP7*	Ubiquitin-Specific Peptidase 7
*PFKM*	Phosphofructokinase, Muscle Type
*CNTN1*	Contactin 1
GLUT3	Glucose transporter type 3
*SLC16A7*	Solute Carrier Family 16 Member 7 (Monocarboxylate Transporter 2)
PD-L1	Programmed death-ligand 1
CAFs	Cancer-Associated Fibroblasts
*LOX*	Lysyl oxidase
TGF-β	Transforming growth factor beta
IL-10	Interleukin 10
Tregs	Regulatory T cells
MDSCs	Myeloid-derived suppressor cells
DCs	Dendritic cells
TAMs	Tumor-associated macrophages
TIME	Tumor Immune Microenvironment
MCTs	Monocarboxylate Transporters
IC50	Half-maximal inhibitory concentration
LC-MS/MS	Liquid Chromatography-Tandem Mass Spectrometry
AUC	Area under the curve
HGLRGs	Hypoxia-, glycolysis-, and lactylation-related genes
IMPS	Immune microenvironment-related prognostic signature
ELISA	Enzyme-Linked Immunosorbent Assay
PLA	proximity ligation assay
scRNA-seq	Single-cell RNA sequencing
